# Three-Dimensional Interferometric ISAR Imaging Algorithm Based on Cross Coherence Processing

**DOI:** 10.3390/s21155073

**Published:** 2021-07-27

**Authors:** Qian Lv, Shaozhe Zhang

**Affiliations:** School of Physics and Information Technology, Shaanxi Normal University, West Chang’an Avenue, No. 620, Xi’an 710119, China; 15565239368@163.com

**Keywords:** 3D imaging, Dechirp processing, interferometric inverse synthetic aperture radar (InISAR), L-shape InISAR system, target reconstruction

## Abstract

Interferometric inverse synthetic aperture radar (InISAR) has received significant attention in three-dimensional (3D) imaging due to its applications in target classification and recognition. The traditional two-dimensional (2D) ISAR image can be interpreted as a filtered projection of a 3D target’s reflectivity function onto an image plane. Such a plane usually depends on unknown radar-target geometry and dynamics, which results in difficulty interpreting an ISAR image. Using the L-shape InISAR imaging system, this paper proposes a novel 3D target reconstruction algorithm based on Dechirp processing and 2D interferometric ISAR imaging, which can jointly estimate the effective rotation vector and the height of scattering center. In order to consider only the areas of the target with meaningful interferometric phase and mitigate the effects of noise and sidelobes, a special cross-channel coherence-based detector (C3D) is introduced. Compared to the multichannel CLEAN technique, advantages of the C3D include the following: (1) the computational cost is lower without complex iteration and (2) the proposed method, which can avoid propagating errors, is more suitable for a target with multi-scattering points. Moreover, misregistration and its influence on target reconstruction are quantitatively discussed. Theoretical analysis and numerical simulations confirm the suitability of the algorithm for 3D imaging of multi-scattering point targets with high efficiency and demonstrate the reliability and effectiveness of the proposed method in the presence of noise.

## 1. Introduction

Due to its superiority in all-day, all-weather and high-resolution applications, inverse synthetic aperture radar (ISAR) has attracted much attention in both civil and military fields [1,2,3,4,5,6]. Typically, the conventional ISAR imaging system can produce a two-dimensional (2D) image of the moving target, which can be interpreted as a filtered projection of the three-dimensional (3D) target’s reflectivity function onto a so-called image projection plane (IPP) [7,8,9,10]. Due to the dependence of the IPP on the target’s own motions and radar-target geometry, such projection cannot be predicted beforehand, which makes it more difficult to interpret ISAR images. A previous attempt to estimate IPP information in advance has been conducted, but its applicability and effectiveness are not confirmed [11]. To radically solve the problem of interpreting 2D ISAR images and support automatic target classification and recognition (ATC/ATR), 3D imaging techniques have emerged and motivated the development of various configurations of 3D ISAR imaging systems [12,13,14,15,16,17,18,19,20,21,22,23,24,25,26,27,28,29,30,31,32,33]. These methods for creating 3D ISAR images can be mainly divided into three different categories.

The first class of method aims at producing 3D target reconstruction by exploiting a single sensor ISAR image sequence [27]. The 3D position of each target’s scattering center is determined by processing the tracks on the ISAR image plane. In this case, knowledge of the relative dynamics corresponding to each ISAR image must be obtained precisely. In addition, this method requires a long target observation time to generate the ISAR image sequence, which is usually uncontrolled and unsatisfactory in a complex imaging environment (such as highly maneuvering airplanes and ships fluctuating with oceanic waves). The second type of approach to create 3D images is based on a 2D antenna array [31,32,33]. Although such an approach circumvents complex motion compensation, it seriously restricts the observation area [28]. In order to acquire fine spatial resolution, a very wide antenna aperture and a large number of antenna elements are necessary, which undoubtedly increases the complexity of the radar system and causes difficulties in signal processing. The third variety of method is interferometric imaging techniques that achieve 3D target reconstruction by combining interferometric processing with ISAR processing [13,14,15,16,17,18,19,20,21,22,23,24,25,26,29,30]. In comparison with the aforementioned methods, both the InISAR system and signal processing are easier to realize. Reported applications of InISAR include target scattering diagnosis, motion modeling, 3D imaging of moving targets, automatic aircraft landing and target recognition [34].

For InISAR imaging, the target geometry is estimated using the interferometric phase of ISAR images acquired from different aperture centers. For this purpose, many 3D InISAR imaging algorithms based on continuous wave (CW) radar have been proposed and applied in practice. In [18], a dual interferometric ISAR system with multichannel CLEAN technique (MC-CLEAN) was utilized to reconstruct 3D images of moving targets. The real data from a suitably designed Multi-Channel ground-based radar was used to verify the reliability of a 3D InISAR technique in [21]. Meanwhile, several successful 3D InISAR algorithms based on a linear frequency modulation (LFM) signal model have also been proposed. In [24], a practical maneuvering target 3D imaging algorithm based on an arbitrary three-antenna InISAR configuration was investigated. To produce high-resolution InISAR images of a maneuvering target, a novel algorithm using the peak extraction technique was proposed in [25]. In the above two references, emphasis was also placed on target motion compensation and motion error, which is of great value for practical applications. In [29,30], time-frequency transform (TFT) and clean technique were used to achieve 3D reconstruction of targets with nonsevere maneuverability. In [23], 3D InISAR imaging combining bistatic configuration with compressive sensing (CS) was proposed, which was especially useful for ship targets, whereas a bistatic radar system on its own was subject to synchronization error.

However, there are several aspects that restrict the applications of the InISAR algorithms above. First, for CW radar, the configurations generally are of one of the following two kinds: (1) three antennas that both transmit and receive, which must utilize orthogonal codes to separate the three channels or (2) one transmitter and a number of collocated receivers. Unfortunately, in either case, such an approach suffers from complex system processing and hardware consumption. Second, in the InISAR imaging system, the multichannel 2D ISAR images are usually utilized to perform interferometric processing. Thus, extraction of the scattering center is carried out using the MC-CLEAN technique, of which the computational cost is at least three times more than that of traditional 2D ISAR imaging [23]. The methods in [18,21,24,25,33,34] all use the CLEAN technique, which is computationally expensive, especially for multi-scattering point targets. Third, the effective interferometric phase provides useful information only in areas with high coherence among three ISAR images [21]. In order to guarantee the precision of parameter estimation, it is crucial to obtain well-focused 2D ISAR images and accurate scattering center extraction. However, some areas without benefit to 3D reconstruction, such as areas with noise and low power areas (in particular secondary lobes from prominent scatters), may also have high coherence, which degrade the quality of the 3D InISAR reconstruction.

To overcome the above drawbacks, this paper presents an InISAR imaging system consisting of two orthogonal baselines based on an LFM signal model, which is more suitable for high resolution imaging purposes [35,36,37,38]. Dechirp processing and Fourier Transformation (FT) are applied to obtain the analytical expression of the complex ISAR image in range frequency-Doppler domain. As Dechirp processing is simple and flexible for hardware, it is now widely applied in ISAR imaging and the radar signal processing field [39]. Finally, the 3D coordinates of the target can be acquired by interferometry and mathematical processing. In order to restrain noise and sidelobes effectively, this paper introduces a detector combining cross-channel coherence with power using three ISAR images. With this method, the interferometric phase of interest can be easily selected and computational cost can be saved by avoiding use of the MC-CLEAN technique. Quantitative analysis of coregistration between two ISAR images from different interferometric antennas for the same baseline is conducted. The simulations are given to verify the proposed algorithm, illustrating that the algorithm is suitable for 3D imaging of multiple scattering points at a low computational cost.

The organization of this paper is as follows. In Section 2, the received signal model is established. The proposed 3D InISAR imaging algorithm is discussed in detail in Section 3. Section 4 gives detailed performance analysis of the proposed algorithm. The simulations of the 3D InISAR imaging algorithm and the conclusions are given in Section 5 and Section 6, respectively.

## 2. Received Signal Model

**Figure 1:**
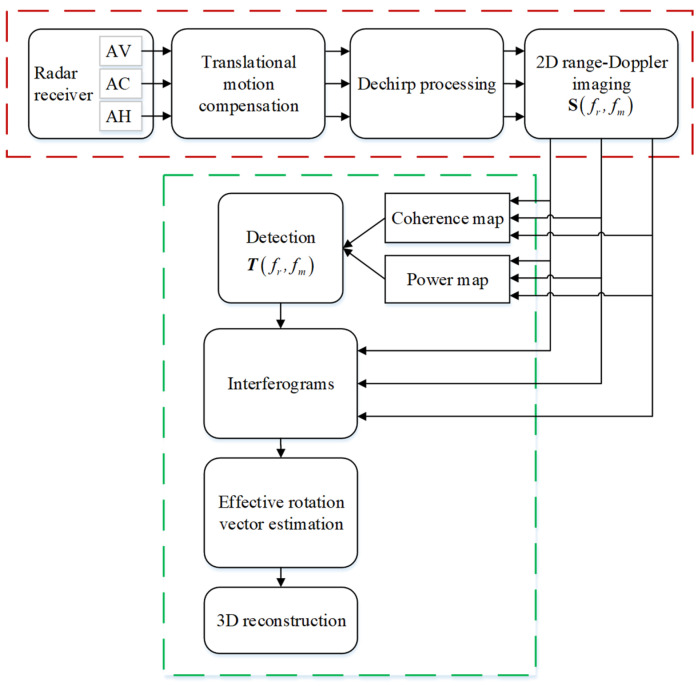
Overall flowchart of 3D target reconstruction with the proposed algorithm.

In this section, to make the proposed 3D reconstruction procedure straightforward, the overall flowchart of the 3D InISAR reconstruction processing is first depicted in Figure 1. Then, the geometry of the system is described, followed by the derivation of the received signal model.

### 2.1. System Geometry

**Figure 2:**
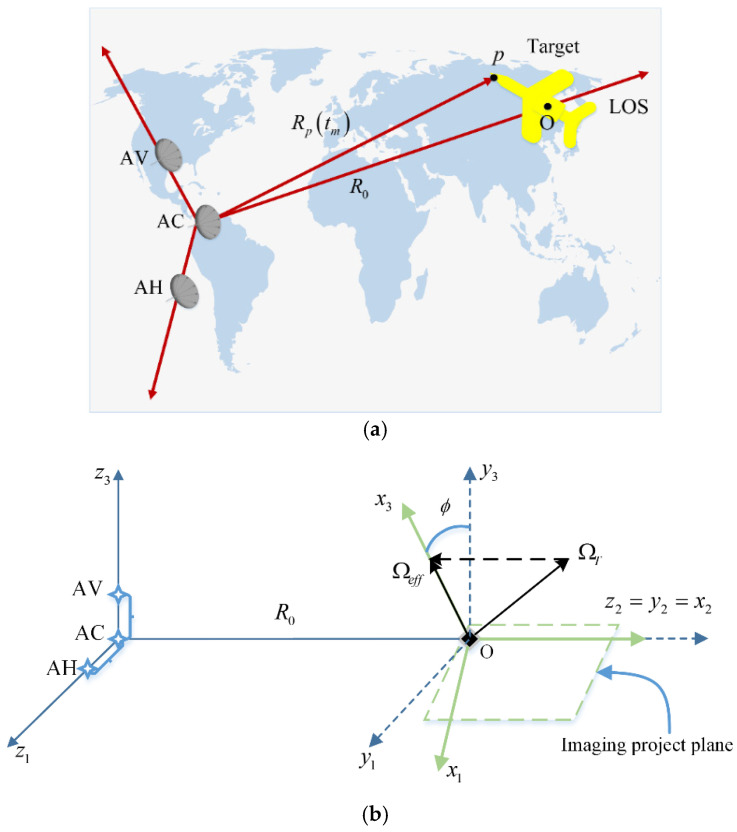
3D InISAR system. (**a**) General scene for InISAR imaging. (**b**) Geometry of radar and target of interest.

In accordance with [18,29], the 3D InISAR imaging system geometry shown in Figure 2a is composed of three receiver antennas labeled AV, AC, and AH, where antenna AC acting as the transmitter is located at the origin of the reference system. In Figure 2b, receivers AV and AH are located on a horizontal and a vertical baseline with coordinates (0,0,dv) and (dh,0,0) respectively. The Cartesian reference system $T_{z}\left(z_{1},z_{2},z_{3}\right)$ is embedded in the radar system with its origin at the array phase center. The axis $z_{2}$ is oriented along the line of sight (LOS) direction from the radar to the center of the target, while $z_{1}$ and $z_{3}$ correspond to the horizontal and vertical baseline respectively. The projection of the total target rotation vector $\Omega_{T}$ onto the plane orthogonal to the LOS defines the effective rotation vector $\Omega_{eff}$. The Cartesian reference system $T_{x}\left(x_{1},x_{2},x_{3}\right)$ is embedded in the target and centered at the focusing point O. Axis $x_{2}$ is oriented along $z_{2}$, whereas $x_{3}$ is oriented along $\Omega_{eff}$. Since the effective rotation vector changes its orientation in time, the reference system $T_{x}$ is time varying. Another reference system $T_{y}$ is defined to be centered on the target with fixed orientation and coincident with $T_{x}$ for slow time $t_{m}=0$. $R_{0}$ is the distance of the radar to the focus center of the target at $t_{m}=0$.

Interestingly, the matrix $M_{zx}$, which describes the rotation of $T_{x}$ with respect to $T_{z}$, is defined as follows:

(1) $$M_{zx}=\left[\begin{array}{ccc} \cos\phi & 0 & \sin\phi \\ 0 & 1 & 0\\ -\sin\phi & 0 & \cos\phi \end{array}\right]$$

Thus, the LOS unit vectors $i_{LOS-x}^{}$ can be written as Equation (2) by means of the rotation matrix $M_{zx}$:

(2) $$i_{LOS-x}^{AV}=\left[\begin{array}{ccc} \frac{-dv\sin\phi }{\sqrt{R_{0}\left(t_{m}\right)+dv^{2}}} & \frac{R_{0}\left(t_{m}\right)}{\sqrt{R_{0}\left(t_{m}\right)+dv^{2}}} & \frac{-dv\cos\phi }{\sqrt{R_{0}\left(t_{m}\right)+dv^{2}}} \end{array}\right]\approx \left[\begin{array}{ccc} \frac{-dv\sin\phi }{R_{0}} & 1 & \frac{-dv\cos\phi }{R_{0}} \end{array}\right]\;i_{LOS-x}^{AC}=\left[\begin{array}{ccc} 0 & 1 & 0 \end{array}\right]\;i_{LOS-x}^{AH}=\left[\begin{array}{ccc} \frac{-dh\cos\phi }{\sqrt{R_{0}\left(t_{m}\right)+dh^{2}}} & \frac{R_{0}\left(t_{m}\right)}{\sqrt{R_{0}\left(t_{m}\right)+dh^{2}}} & \frac{dh\sin\phi }{\sqrt{R_{0}\left(t_{m}\right)+dh^{2}}} \end{array}\right]\approx \left[\begin{array}{ccc} \frac{-dh\cos\phi }{R_{0}} & 1 & \frac{dh\sin\phi }{R_{0}} \end{array}\right]$$

where $R_{0}\left(t_{m}\right)\approx R_{0}$ is valid in a far field condition, so the terms $\sqrt{R_{0}+dv^{2}}$ and $\sqrt{R_{0}+dh^{2}}$ are approximated as $R_{0}$ for a small observation time. $y=\left[y_{1},y_{2},y_{3}\right]$ denotes the position for a generic scatterer at $t_{m}=0$ in $T_{y}$.

Under the assumption of a small observation time value $T_{a}$, the total rotation vector $\Omega_{T}$ can be considered as constant and the image plane as fixed with respect to $T_{z}$ [1,2,3,4,5,6,7,8,9,10,11,12,13,14,15,16,17,18,19]. Therefore, $x\left(t_{m}\right)$, the location of an arbitrary scatterer at a time instant $t_{m}$ in $T_{x}$, can be expressed in a closed form by solving the differential equation system $\dot{x}\left(t_{m}\right)=\Omega_{T}\times x\left(t_{m}\right)$ with initial condition x(0)=y. The result is as follows:

(3) $$x\left(t_{m}\right)=a+b\cos\left(\Omega_{T}t_{m}\right)+\frac{c}{\Omega_{T}}\sin\left(\Omega_{T}t_{m}\right)$$

where $\Omega_{T}=\left|\Omega_{T}\right|,a=\frac{\Omega_{T}y}{\Omega_{T}{}^{2}}\Omega_{T},b=y-\frac{\Omega_{T}y}{\Omega_{T}{}^{2}}\Omega_{T},c=\Omega_{T}\times y$.

For values of the observation time lower than 1s, the term $x\left(t_{m}\right)$ in Equation (3) can be reasonably approximated by its first-order Taylor series at $t_{m}=0$ [1,2,3,4,5,6,7,8,9,10,11,12,13,14,15,16,17,18]:

(4) $$x\left(t_{m}\right)=y+ct_{m}$$

where $c=\left[c_{1},c_{2},c_{3}\right]$.

As a consequence, the distance sum $\Delta R_{p}\left(t_{m}\right)={\left[\Delta R_{p}^{AV}\left(t_{m}\right),\Delta R_{p}^{AC}\left(t_{m}\right),\Delta R_{p}^{AH}\left(t_{m}\right)\right]}^{T}$ between the focusing point O and the projection of the scatterer on the LOS in the different channels (AV, AC, and AH) can be written as follows:

(5) $$\Delta R_{p}\left(t_{m}\right)=\left(i_{LOS-x}^{AC}+i_{LOS-x}^{A\Gamma }\right)x{\left(t_{m}\right)}^{T}=K_{0}^{}+K_{1}^{}t_{m}$$

where $K_{0}^{}={\left[K_{0}^{AV},K_{0}^{AC},K_{0}^{AH}\right]}^{T}$, $K_{1}^{}={\left[K_{1}^{AV},K_{1}^{AC},K_{1}^{AH}\right]}^{T}$ and

$$K_{0}^{AV}=2y_{2}-\frac{dv}{R_{0}}\left(y_{1}\sin\phi +y_{3}\cos\phi \right),\;K_{1}^{AV}=2c_{2}-\frac{dv}{R_{0}}\left(c_{1}\sin\phi +c_{3}\cos\phi \right)\;K_{0}^{AC}=2y_{2},\;K_{1}^{AC}=2c_{2}\;K_{0}^{AH}=2y_{2}-\frac{dh}{R_{0}}\left(y_{1}\cos\phi -y_{3}\sin\phi \right),\;K_{1}^{AH}=2c_{2}-\frac{dh}{R_{0}}\left(c_{1}\cos\phi -c_{3}\sin\phi \right)$$

### 2.2. Received Signal Modeling

Assume that the radar transmits a normalized LFM signal, which takes the form of:

(6) $$s_{t}\left(\hat{t},t_{m}\right)=rect\left(\frac{\hat{t}}{T_{s}}\right)\exp\left(j2\pi \left(f_{c}\hat{t}+\frac{1}{2}\gamma {\hat{t}}^{2}\right)\right),$$

where $\hat{t}$ is the fast time, $T_{s}$ is the pulse duration, $f_{c}$ is the carrier frequency, γ is the frequency modulation rate, and rect() denotes a unit rectangle function. $t_{m}=mT_{r}\left(m=0,1,2\ldots \right)$ is the slow time, where $T_{r}$ is the pulse repetition period. The complex envelope of the received signals from the pth scattering center on the target is given as follows:

(7) sr(t^,tm)=δprect(t^−Rp(tm)cTs)exp[j2πfc(t^−Rp(tm)c)]exp[jπγ(t^−Rp(tm)c)2]

where $s_{r}\left(\hat{t},t_{m}\right)={\left[s_{r}^{AV}\left(\hat{t},t_{m}\right),s_{r}^{AC}\left(\hat{t},t_{m}\right),s_{r}^{AH}\left(\hat{t},t_{m}\right)\right]}^{T}$ is expressed by means of a multichannel vector. When the baselines are short compared with the radar–target distance, $\delta_{p}$ can be considered as the same for channels AV, AC, and AH. c is the speed of light, and $R_{p}\left(t_{m}\right)={\left[R_{p}^{AV}\left(t_{m}\right),R_{p}^{AC}\left(t_{m}\right),R_{p}^{AH}\left(t_{m}\right)\right]}^{T}$ denotes the sum of the distance from the pth scattering center to the transmitter (AC) and the receivers (AV, AC, AH).

As demonstrated in [40], the signal from the focus center of the ISAR image is selected as the reference signal to Dechirp the received signal at the receivers AV, AC, and AH. The reference signal from the reference point can be expressed as:

(8) $$s_{ref}\left(\hat{t},t_{m}\right)=rect\left(\frac{\hat{t}-R_{ref}/c}{T_{ref}}\right)exp\left[j2\pi f_{c}\left(\hat{t}-\frac{R_{ref}}{c}\right)\right]exp\left[j\pi \gamma {\left(\hat{t}-\frac{R_{ref}}{c}\right)}^{2}\right]$$

where $T_{ref}$ is the duration of the reference signal and $T_{ref}>T_{s}$, $R_{ref}\approx 2R_{0}$. The received signal after dechirping can be expressed as in [40]:

(9) $$s_{if}\left(\hat{t},t_{m}\right)=s_{r}\left(\hat{t},t_{m}\right)\times {\left[s_{ref}\left(\hat{t},t_{m}\right)\right]}^{*}$$

where * denotes the conjugation operation, and $s_{ref}\left(\hat{t},t_{m}\right)={\left[s_{ref}^{AV}\left(\hat{t},t_{m}\right),s_{ref}^{AC}\left(\hat{t},t_{m}\right),s_{ref}^{AH}\left(\hat{t},t_{m}\right)\right]}^{T}$. In order to obtain well-focused images for moving targets, motion compensation is required for 2D ISAR imaging. However, if the translational motion compensation is made for three channels independently, the ISAR images obtained from three channels will have different focus centers, which may result in pixel misregistration and great difficulties in scattering center extraction. Discussion of coherent translational motion compensation is outside the scope of this paper, and the reader can refer to [41,42] for more details. Assuming that coherent translational motion compensations have been completed, and thus only focusing on the contribution of the rotational motion to ISAR imaging, the signal in Equation (9) can be represented as:

(10) $$\begin{array}{c} s_{if}\left(\hat{t},t_{m}\right)=\delta_{p}rect\left(\frac{\hat{t}-R_{ref}/c}{T_{ref}}\right)exp\left[-j\frac{2\pi \gamma }{c}\left(\hat{t}-\frac{R_{ref}}{c}\right)\Delta R_{p}\left(t_{m}\right)\right] \end{array}\;\times exp\left[-j\frac{2\pi f_{c}}{c}\Delta R_{p}\left(t_{m}\right)\right]exp\left[j\frac{\pi \gamma }{c^{2}}{\left(\Delta R_{p}\left(t_{m}\right)\right)}^{2}\right]$$

Taking the time of the reference point as a basis, fast Fourier transform (FFT) is performed on Equation (10) with respect to $\hat{t}$, obtaining:

(11) $$\begin{array}{c} S_{IF}\left(f_{r},t_{m}\right)=\delta_{p}T_{s}\mathrm{sinc}\left[T_{s}\left(f_{r}+\frac{\gamma }{c}\Delta R_{p}\left(t_{m}\right)\right)\right]\exp\left(-j\frac{2\pi f_{c}}{c}\Delta R_{p}\left(t_{m}\right)\right) \end{array}\;\times \exp\left(-j\frac{2\pi f_{r}}{c}\Delta R_{p}\left(t_{m}\right)\right)\exp\left(-j\frac{\pi \gamma }{c^{2}}{\left(\Delta R_{p}\left(t_{m}\right)\right)}^{2}\right)$$

where $S_{IF}\left(f_{r},t_{m}\right)={\left[S_{IF}^{AV}\left(f_{r},t_{m}\right),S_{IF}^{AC}\left(f_{r},t_{m}\right),S_{IF}^{AH}\left(f_{r},t_{m}\right)\right]}^{T}$, $f_{r}$ denotes the range frequency. As Equation (11) demonstrates, the energy of the received signal peaks along the line $f_{r}=-\frac{\gamma }{c}\Delta R_{p}\left(t_{m}\right)$ in the frequency domain. After envelope alignment and removing the residual video phase (RVP) by using the compensation function $S_{C}\left(f_{r},t_{m}\right)=\exp\left(-j\pi f_{r}{}^{2}/\gamma \right)$, the multichannel received signal can be rewritten as follows [40]:

(12) $$\begin{array}{c} S\left(f_{r},t_{m}\right)=S_{IF}\left(f_{r},t_{m}\right)S_{C}\left(f_{r},t_{m}\right) \end{array}\;=\delta_{p}T_{s}\sin c\left[T_{s}\left(f_{r}+\frac{\gamma }{c}K_{0}^{}\right)\right]\exp\left[-j\frac{2\pi f_{c}}{c}\left(K_{0}^{}+K_{1}^{}t_{m}\right)\right]$$

It can be seen from Equation (12) that the radial distance of different scattering centers on the target corresponds with the different frequency range of the received signal after Dechirping. Therefore, through frequency analysis, accurate resolution in the range dimension can be achieved. Similarly, by performing FFT on Equation (12) with respect to slow time $t_{m}$, the received signal in the range frequency–Doppler domain can be written as follows:

(13) $$S\left(f_{r},f_{m}\right)=\delta_{p}T_{s}T_{a}\exp\left[-j\frac{2\pi }{\lambda }K_{0}^{}\right]\times \sin c\left[T_{s}\left(f_{r}+\frac{\gamma }{c}K_{0}^{}\right)\right]\times \sin c\left[T_{a}\left(f_{m}+\frac{K_{1}^{}}{\lambda }\right)\right]$$

where $f_{m}$ denotes the Doppler frequency. In Equation (13), if the same scatterer appears in the same pixel in all three images, the interferometric phase can be calculated directly without need for the coregistration algorithm, which will be analyzed in Section 4.

## 3. 3D Target Reconstruction Based on the Proposed Algorithm

In Section 2, three 2D ISAR images are obtained by Dechirp processing and a FFT operation. In this section, a novel ISAR imaging algorithm is proposed to reconstruct the 3D target image, which significantly reduces computational cost and avoids image misregistration. The main steps of the proposed algorithm include: (i) obtaining three channel ISAR images with basic signal processing, such as motion compensation and pulse compression by Dechirping and a FFT operation; (ii) applying a cross-channel coherence technique to extract effective scattering center among three ISAR images; (iii) estimating the effective rotation vector $\Omega_{eff}$ and reconstructing the 3D ISAR image.

### 3.1. Scattering Center Extraction via Cross-Channel Coherence-Based Detector

By considering the received signal model in Section 2, in order to make the following analysis clearer, the ISAR images from the three channels in Equation (13) can be rewritten as:

(14) $$S\left(f_{r},f_{m}\right)=\{\begin{array}{l} S_{}^{AV}\left(f_{r},f_{m}\right)=\delta_{p}T_{s}T_{a}\exp\left[-j\frac{2\pi }{\lambda }K_{0}^{AV}\right]\times \sin c\left[T_{s}\left(f_{r}+\frac{\gamma }{c}K_{0}^{AV}\right)\right]\times \sin c\left[T_{a}\left(f_{m}+\frac{K_{1}^{AV}}{\lambda }\right)\right]\\ S_{}^{AC}\left(f_{r},f_{m}\right)=\delta_{p}T_{s}T_{a}\exp\left[-j\frac{2\pi }{\lambda }K_{0}^{AC}\right]\times \sin c\left[T_{s}\left(f_{r}+\frac{\gamma }{c}K_{0}^{AC}\right)\right]\times \sin c\left[T_{a}\left(f_{m}+\frac{K_{1}^{AC}}{\lambda }\right)\right]\\ S_{}^{AH}\left(f_{r},f_{m}\right)=\delta_{p}T_{s}T_{a}\exp\left[-j\frac{2\pi }{\lambda }K_{0}^{AH}\right]\times \sin c\left[T_{s}\left(f_{r}+\frac{\gamma }{c}K_{0}^{AH}\right)\right]\times \sin c\left[T_{a}\left(f_{m}+\frac{K_{1}^{AH}}{\lambda }\right)\right] \end{array}$$

Therefore, the phase differences at the peaks of the sinc function can be computed by exploiting the received signal in Equation (14) as follows:

(15) $$\begin{array}{c} \Delta \vartheta_{AV}=∠\left(S_{}^{AV}\left(f_{r},f_{m}\right)S^{AC}{}^{*}\left(f_{r},f_{m}\right)\right)\\ \Delta \vartheta_{AH}=∠\left(S_{}^{AH}\left(f_{r},f_{m}\right)S^{AC}{}^{*}\left(f_{r},f_{m}\right)\right) \end{array}$$

It is known that noise and secondary lobes from prominent scatters influence the interferometric phase of interest. In order to remove these effects, thresholds in power and coherence are jointly used to detect the areas of interest. Normally, only when image pixels exceed both the coherence threshold and the power threshold will the areas be extracted to form the final InISAR image.

The cross-channel coherence among three ISAR images for each resolution cell is computed as follows:

(16) $$C^{ij}\left(f_{r},f_{m}\right)=\frac{\left|S_{}^{i}\left(f_{r},f_{m}\right)S_{}^{j}{\left(f_{r},f_{m}\right)}^{*}\right|}{\sqrt{{\left|S_{}^{i}\left(f_{r},f_{m}\right)\right|}^{2}{\left|S_{}^{j}\left(f_{r},f_{m}\right)\right|}^{2}}},\;\forall i\neq j$$

where i=1,2,3 denotes channels AV, AC, and AH respectively. In addition, it can be seen that:

(17) $$P^{i}\left(f_{r},f_{m}\right)=\left|S_{}^{i}\left(f_{r},f_{m}\right)\right|$$

Therefore, an effective detection algorithm, known as a cross-channel coherence-based detector (C3D), is defined by:

(18) $$T\left(f_{r},f_{m}\right)=\left[\overset{3}{\underset{i=1,i\neq j}{\prod }}C^{ij}\left(f_{r},f_{m}\right)>C_{th}\right]\&\left[\overset{3}{\underset{i=1}{\prod }}P^{i}\left(f_{r},f_{m}\right)>P_{th}\right]$$

where & denotes Boolean product. Thus, extraction of the scattering center from an ISAR image can be performed with such a detection mask. Only image pixels exceeding the coherence threshold $C_{th}$ and power threshold $P_{th}$ will contribute to the 3D target reconstruction. Therefore, for each ISAR image, a noise area where the target of interest does not appear can be selected to estimate the noise level $P_{\mathrm{noise}}$. The detection threshold can be obtained by estimating the noise level of each ISAR image. For single channel ISAR image detection, we can set $P_{\sin\mathrm{gle}}=P_{\mathrm{noise}}+15\;\mathrm{dB}$, and the coherence threshold would be prepared in a similar way to the power threshold [21].

### 3.2. 3D Target Reconstruction via Joint Estimation of $\Omega_{eff}$

It is observed from Equations (14) and (15) that the phase differences at the peak of the sinc function for horizontal and vertical configurations can be computed as:

(19) $$\Delta \vartheta_{AV}=∠\left(S^{AV}\left(f_{r},f_{m}\right)S^{AC}{}^{*}\left(f_{r},f_{m}\right)\right)=\frac{2\pi }{\lambda }\left(K_{0}^{AC}-K_{0}^{AV}\right)=\frac{2\pi }{\lambda }\frac{dv}{R_{0}}\left(y_{1}\sin\phi +y_{3}\cos\phi \right)\;\Delta \vartheta_{AH}=∠\left(S^{AH}\left(f_{r},f_{m}\right)S^{AC}{}^{*}\left(f_{r},f_{m}\right)\right)=\frac{2\pi }{\lambda }\left(K_{0}^{AC}-K_{0}^{AH}\right)=\frac{2\pi }{\lambda }\frac{dh}{R_{0}}\left(y_{1}\cos\phi -y_{3}\sin\phi \right)$$

In other words, the coordinates of each scatterer can be expressed as a function of the phase differences. Particularly, the component $y_{3}$ denotes the height of the scatterer with respect to the image plane. From this, we can proceed to:

(20) $$\begin{array}{c} y_{1}=\frac{\lambda R_{0}}{2\pi }\left(\frac{\Delta \vartheta_{AV}}{dv}\sin\phi +\frac{\Delta \vartheta_{AH}}{dh}\cos\phi \right)\\ y_{3}=\frac{\lambda R_{0}}{2\pi }\left(\frac{\Delta \vartheta_{AV}}{dv}\cos\phi -\frac{\Delta \vartheta_{AH}}{dh}\sin\phi \right) \end{array}$$

Furthermore, with respect to $T_{x}$, the term c can be represented as:

(21) $$c=\Omega_{T}\times y\Rightarrow \left[c_{1},c_{2},c_{3}\right]=\left|\begin{array}{c} \begin{array}{ccc} 0 & \Omega_{T2} & \Omega_{eff} \end{array}\\ \begin{array}{ccc} y_{1} & y_{2} & y_{3} \end{array} \end{array}\right|\Rightarrow c_{2}=y_{1}\Omega_{eff}$$

where $\Omega_{eff}$ is the modulus of $\Omega_{eff}$. It should be noted that the term $c_{2}$ can be estimated by considering the Doppler component measured from the AC channel:

(22) $$f_{m}^{AC}=-\frac{K_{1}^{AC}}{\lambda }=-\frac{2c_{2}}{\lambda }$$

Substituting Equations (20) and (21) into Equation (22) yields:

(23) $$f_{m}^{AC}=-\frac{R_{0}}{\pi }\left(\frac{\Delta \vartheta_{AV}}{dv}\Omega_{eff}\sin\phi +\frac{\Delta \vartheta_{AH}}{dh}\Omega_{eff}\cos\phi \right)$$

It is worth noting that the unknown parameters $\Omega_{eff}$ and ϕ can be jointly estimated using Equation (23). For simplicity, Equation (23) can be rewritten by defining the following quantities:

(24) $$\begin{array}{c} Z=f_{m}^{AC},Y=-\frac{R_{0}}{\pi }\frac{\Delta \vartheta_{AV}}{dv},X=-\frac{R_{0}}{\pi }\frac{\Delta \vartheta_{AH}}{dh},\\ \alpha =\Omega_{eff}\sin\phi ,\beta =\Omega_{eff}\cos\phi . \end{array}$$

If there are a total of P scatterers extracted from the ISAR images, Equation (24) can be rewritten as:

(25) Z=αY+βX

where $Z={\left[Z_{1},Z_{2},\ldots ,Z_{P}\right]}^{T}$ represents the Doppler information of the extracted scatterers for the central channel, $Y={\left[Y_{1},Y_{2},\ldots ,Y_{P}\right]}^{T}$ and $X={\left[X_{1},X_{2},\ldots ,X_{P}\right]}^{T}$ correspond to the vectors related to the interferometric phase matrices $\Delta \vartheta_{AV}$ and $\Delta \vartheta_{AH}$ only in the effective range and Doppler cells where the scatterers are extracted. In this respect, the elements in terms Z, Y and X are real values and Equation (25) denotes the equation of a plane, which can be done by evaluating the regression plane.

Consequently, the two parameters $\Omega_{eff}$ and ϕ can be estimated by first estimating α and β, and this problem can be solved by minimizing the function:

(26) $$ℜ\left(\alpha ,\beta \right)=\overset{P}{\underset{p=1}{\sum }}\left[Z_{p}-\left(\alpha Y_{p}+\beta X_{p}\right)\right]$$

Finally, the estimation of $\Omega_{eff}$ and ϕ can be calculated from the estimation of the estimated $\hat{\alpha }$ and $\hat{\beta }$ as described by:

(27) $${\hat{\Omega }}_{eff}=\sqrt{{\hat{\alpha }}^{2}+{\hat{\beta }}^{2}},\;\hat{\phi }=a\tan\left(\frac{\hat{\alpha }}{\hat{\beta }}\right)$$

## 4. Performance Analysis of the Proposed Algorithm

In Section 3, a 3D InISAR reconstruction algorithm with Dechirping processing is proposed. There are three crucial points to guarantee good reconstruction performance, namely image registration, computational cost, and soft assignment. In order to accurately define and evaluate the estimation and reconstruction errors, a simple soft assignment method is performed to assign each scatterer of the reconstructed target to the corresponding scatterer of the model.

### 4.1. Mismatch Problem of Image Registration

In an InISAR imaging system, the interferometric processing among three ISAR images is based on accurate image registration. Due to the symmetry of the AV and AH channels, this paper takes the AV and AC receiver pair as an example to research the mismatching of two ISAR images.

Compared to Equation (14), the pixel positions of the pth scatterer in the 2D ISAR images from the receivers AV and AC are:

(28) $$\left(-\frac{\gamma }{c}K_{0}^{AV},-\frac{1}{\lambda }K_{1}^{AV}\right)=\{-\frac{\gamma }{c}\left(2y_{2}-\frac{dv}{R_{0}}\left(y_{1}\sin\phi +y_{3}\cos\phi \right)\right),-\frac{1}{\lambda }\left(2c_{2}-\frac{dv}{R_{0}}\left(c_{1}\sin\phi +c_{3}\cos\phi \right)\right)\}\;\left(-\frac{\gamma }{c}K_{0}^{AC},-\frac{1}{\lambda }K_{1}^{AC}\right)=\left\{-\frac{\gamma }{c}\left(2y_{2}\right),-\frac{1}{\lambda }\left(2c_{2}\right)\right\}$$

From Equation (28), it can be found that the baseline and direction of the effective rotation vector directly result in mismatches in the range frequency–Doppler domain. The range frequency offset in the range direction is:

(29) $$\Delta f_{r}=-\frac{\gamma }{c}\left(K_{0}^{AV}-K_{0}^{AC}\right)=\frac{\gamma }{c}\frac{dv}{R_{0}}\left(y_{1}\sin\phi +y_{3}\cos\phi \right)$$

Thus, in a pulse repetition period, the number of the range resolution cell corresponding to $\Delta f_{r}$ is:

(30) $$\Delta n=\Delta f_{r}T_{s}=\frac{B}{c}\frac{dv}{R_{0}}\left(y_{1}\sin\phi +y_{3}\cos\phi \right)$$

where B stands for the bandwidth of the transmitted signal.

Equally, the Doppler offset in the cross-range direction is:

(31) $$\Delta f_{m}=-\frac{1}{\lambda }\left(K_{1}^{AV}-K_{1}^{AC}\right)=\frac{1}{\lambda }\frac{dv}{R_{0}}\left(c_{1}\sin\phi +c_{3}\cos\phi \right)$$

Thus, in ISAR imaging observation time, the number of the cross-range resolution cell corresponding to $\Delta f_{m}$ is:

(32) $$\Delta m=\Delta f_{m}T_{a}=\frac{T_{a}}{\lambda }\frac{dv}{R_{0}}\left(c_{1}\sin\phi +c_{3}\cos\phi \right)$$

Based on the above analysis, the offset between two ISAR images from different interferometric receivers is correlated with the initial distance, baseline length, target’s rotation, and other parameters. Therefore, according to the geometric relationship between the radar and the target, a quantitative analysis of the offset is given below.

For convenience, the parameters in this paper are utilized to evaluate the image mismatch. Suppose that in a far-field condition, $R_{0}=10\;\mathrm{km}$, dv=dh=1 m, B=200 Hz, $f_{c}=10\;\mathrm{GHz}$, $T_{a}=0.5\;s,\;\phi =45^{\circ }$, and the target size does not exceed 60 m. In this condition:

(33) $$\begin{array}{c} \Delta n=\frac{B}{c}\frac{dv}{R_{0}}\left(y_{1}\sin\phi +y_{3}\cos\phi \right)<1\\ \Delta m=\frac{T_{a}}{\lambda }\frac{dv}{R_{0}}\left(c_{1}\sin\phi +c_{3}\cos\phi \right)<1 \end{array}$$

where $c=\left[c_{1},c_{2},c_{3}\right]$ is related to the total target rotation vector $\Omega_{T}$, which will not be too large [43].

Obviously, in a far-field condition, image misregistration does not appear after Dechirp processing and Fourier transformation in the range frequency–Doppler dimension. However, the mismatch problem in InISAR imaging may occur when the baseline is too long or the target’s size is too large. In this case, some image registration methods are needed to solve or mitigate the misregistration, which is beyond the scope of this paper.

### 4.2. Analysis of the Computational Cost

Based on the performance analysis above, the computational burden for 3D target reconstruction mainly results from dominant scatterer extraction. Here, assume that the number of the resolution cell in the range frequency–Doppler dimension and the number of the scatterers on the target are N and P, respectively.

For a 3D InISAR imaging algorithm based on the joint cross modified Wigner–Ville distribution (jc-MWVD) in [30], its implementation procedure in a certain range cell included the defined symmetric cross-correlation function $\left[N^{2}\right]$, scaled transform $\left[3N^{2}log_{2}N\right]$, and Fourier transform $\left[N^{2}log_{2}N\right]$. Obviously, the computational cost of the algorithm proposed in [30] is on the order of $\left[\left(N^{2}+4N^{2}log_{2}N\right)P\right]$.

For the method in [29], due to the application of the searching procedure, the computational cost is on the order of $\left[\left(N^{2}+MNlog_{2}N\right)P\right]$, where M is the number of searching points and always greater than N for high-resolution imaging [6]. In this method, the searching steps and initial parameters are difficult to control. Therefore, this method is less suitable for realistic application.

In [18], the MC-CLEAN technique is based on the optimization problem and iteration, so the computational cost will increase linearly with the total number of scatterers. As the number of the scatterers on the target (such as an airplane or ship) may be up to hundreds in practice, this may result in a high computational burden.

Although the method in [21] does not use a searching procedure with respect to scattering center extraction, this method still requires approximately $\left[\left(6N^{2}\left(N_{p}^{3}+1\right)+5N^{2}\right)\right]$ multiplication operations due to a $N_{p}\times N_{p}$ moving window.

The proposed C3D only employs multiply–accumulate operations (MAs) to extract the dominant scatterers. The procedure for cross-channel coherence among three ISAR images includes $\left[12N^{2}\right]$ MAs in Equations (16) and (17). The mask in Equation (18) requires $\left[5N^{2}\right]$ MAs. The computational cost is on the order of $\left[12N^{2}+5N^{2}\right]$. More importantly, the performance of the C3D is not affected by the total number of scatterers.

**Figure 3:**
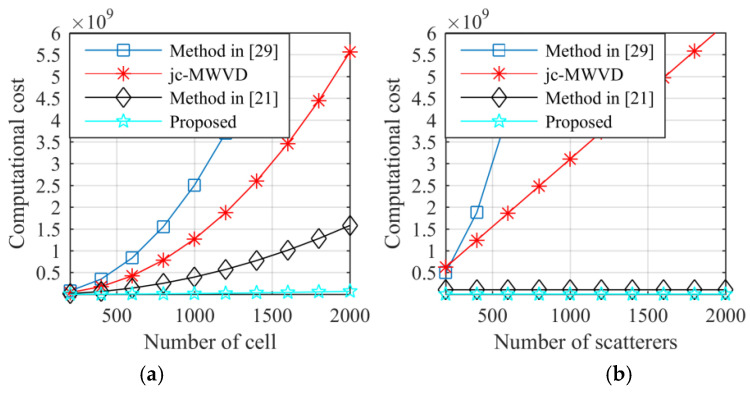
Computational cost comparison. (**a**) Computational cost versus resolution cell (P=31). (**b**) Computational cost versus total scatterers (N=512).

Under the assumptions of $M=8N,N_{p}=4$, Figure 3 shows the computational complexities of the proposed method and three other methods. From Figure 3a, it can be observed that the computational costs of the three other methods increases rapidly with increases in the number of the resolution cell in the range frequency–Doppler dimension. Meanwhile, compared with the method in [29] and the jc-MWVD in Figure 3b, the computational cost of the proposed method is independent of the total number of scatterers, which makes this method better than the other methods of multi-scatterer target imaging. Based on Figure 3, it can be concluded that the proposed method is more computationally efficient and has a wider applicability than the other three methods in ISAR imaging applications.

### 4.3. Soft Assignment Method

In order to assign each scatterer of the 3D reconstructed target to the corresponding scatterer of the model, a soft assignment method based on the probabilistic least squares (PLS) approach [44] is proposed. Accordingly, the soft assignment matrix of dimensions P×K can be defined by:

(34) $$\kappa =\left[\begin{array}{ccc} \kappa_{1,1} & \cdots & \kappa_{1,K}\\ \begin{array}{c} \vdots \\ \vdots \\ \vdots \end{array} & \begin{array}{c} \vdots \\ \kappa_{p,k}\\ \vdots \end{array} & \begin{array}{c} \vdots \\ \vdots \\ \vdots \end{array}\\ \kappa_{P,1} & \cdots & \kappa_{P,K} \end{array}\right]$$

where P and K are the number of the scatterers in the model and the number of the extracted scatterers, respectively. Each element of the matrix $\kappa_{p,k}$ denotes the probability that the kth extracted scatterer belongs to the pth scatterers of the model. The sum of all possible assignments for a given scatterer is equal to the unity. The solution for the soft assignment problem is given as follows:

(35) $$\kappa_{p,k}=\frac{{\left(\varepsilon_{p,k}^{T}\varepsilon_{p,k}^{}\right)}^{-1}}{\sum_{l=1}^{P}{\left(\varepsilon_{l,k}^{T}\varepsilon_{l,k}^{}\right)}^{-1}},\overset{P}{\underset{p=1}{\sum }}\kappa_{p,k}=\forall k=1,\ldots K$$

where $\varepsilon_{p,k}^{}$ is the Euclidean distance between the pth scatterer of the model and the kth extracted scatterer of the reconstructed target.

Therefore, each extracted scatterer can be assigned to the scatterer of the reference model with highest probability. In order to guarantee the precision of soft assignment, we can easily distinguish the scatterer of the reference model and the matching one by setting the appropriate probability threshold $\Lambda_{\kappa }$. In this way, the corresponding height and distance errors between the reference model and the reconstructed target, which are used to evaluate the performance of the proposed algorithm, can be calculated. Since the sum of all possible assignments for a given scatterer adds to the unity, the maximum value of each column is used here as the assignment indicator, namely $\Lambda_{\kappa }=\max\left[\kappa_{k}\right],k=1\ldots K$. In real world applications, when signal-to-noise ratio (SNR) is very low, each value of $\kappa_{k}$ tends to be decentralized, which is detrimental to soft assignment.

## 5. Simulation Results

In this section, simulated radar data will be adopted to verify the effectiveness of the proposed 3D InISAR imaging algorithm. To simulate a given SNR, Gaussian noise is added to the raw data (in the data domain). Several simulations were run to analyze the performance of our target reconstruction method.

### 5.1. Verification with Simple Airplane Model

**Figure 4:**
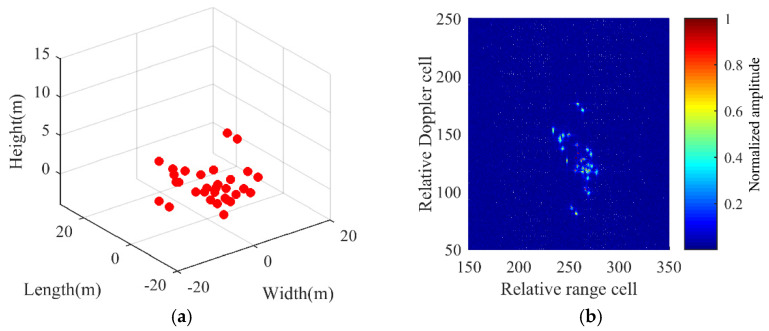
3D airplane model and ISAR image. (**a**) 3D airplane model. (**b**) Traditional 2D ISAR imaging result.

The simulated 3D model of the airplane target, which is composed of P=31 ideal scatterers, is shown in Figure 4a. The simulation parameters are listed in Table 1. Based on the traditional range frequency–Doppler (RD) algorithm in Equation (14), Figure 4b gives the 2D ISAR imaging result from central channel AC.

**Figure 5:**
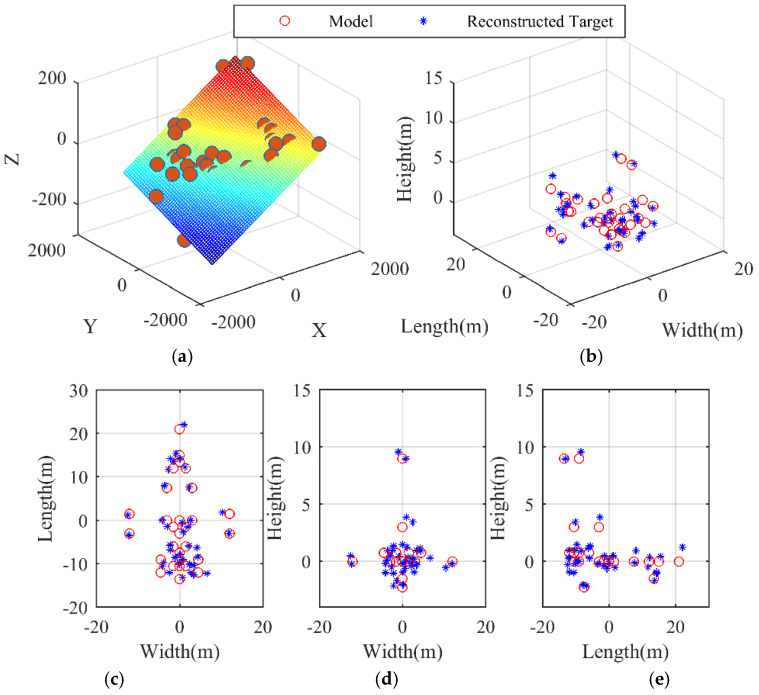
3D reconstruction results dv,dh=0.5 m. (**a**) Regression plane of parameter estimation in Equation (25). (**b**) 3D reconstruction result of the airplane. (**c**) Range–horizontal direction view ($y_{1}-y_{2}$). (**d**) Range–vertical direction view ($y_{2}-y_{3}$). (**e**) Vertical–horizontal direction view ($y_{1}-y_{3}$).

**Figure 6:**
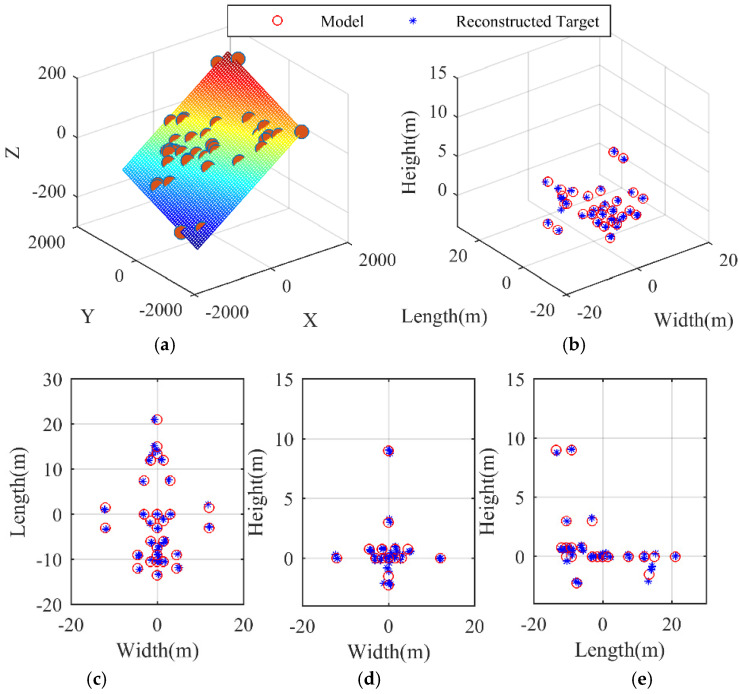
3D reconstruction results dv,dh=4 m. (**a**) Regression plane of parameter estimation in Equation (25). (**b**) 3D reconstruction result of the airplane. (**c**) Range–horizontal direction view ($y_{1}-y_{2}$). (**d**) Range–vertical direction view ($y_{2}-y_{3}$). (**e**) Vertical–horizontal direction view ($y_{1}-y_{3}$).

Figure 5 and Figure 6 provide a clearer and simpler visualization, in which the positions of the scatterers on the airplane are depicted in the planes $y_{1}-y_{2}$, $y_{2}-y_{3}$, and $y_{1}-y_{3}$. The reconstructed target is superimposed onto the model so as to present a more direct viewing comparison. The regression planes of parameter estimation in Equation (25) are shown in Figure 5a and Figure 6a, from which the estimates of $\Omega_{eff}$ and ϕ can be indirectly obtained by estimating the coefficients of the multiple linear regression [45]. In Figure 5b and Figure 6b, it is evident by visual inspection that the 3D targets have been reconstructed, while, for baseline dv=dh=0.5 m, the reconstruction result in Figure 5b is not as good as that in Figure 6b, where the baseline length is 4 m and the reconstructed target matches the reference model perfectly. Below, the performance of the proposed algorithm is analyzed with respect to different SNR and different baseline length.

**Figure 7:**
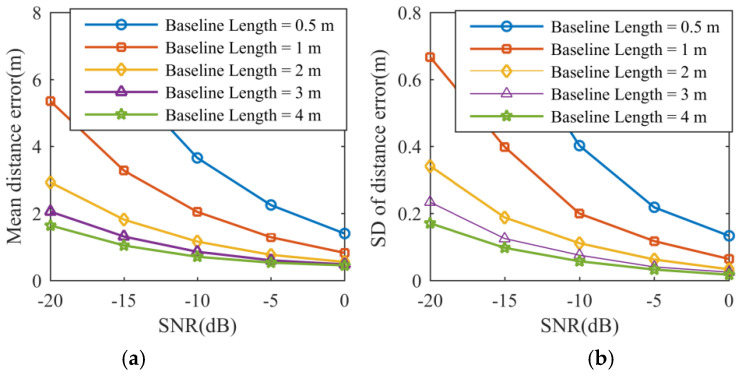
Simulation result. (**a**) Mean distance error. (**b**) Standard deviation of distance error.

**Figure 8:**
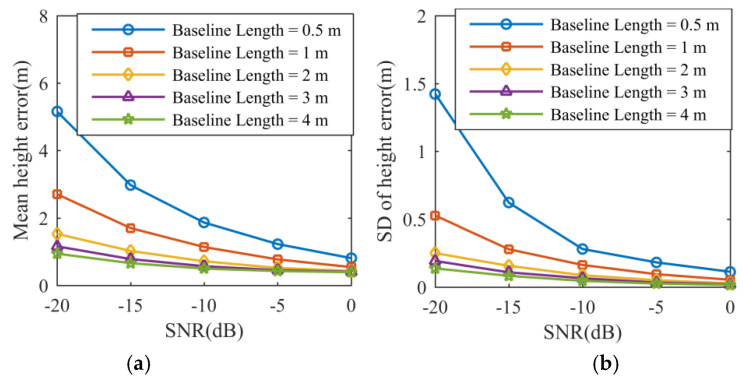
Simulation result. (**a**) Mean distance error. (**b**) Standard deviation of distance error.

In the given scenarios, Figure 7 shows the behaviors of the mean distance error and its standard deviation (SD) expressed in meters with respect to the SNR and the baseline length. The same performance analysis for height is depicted in Figure 8. In particular, the algorithm performance for five different baseline lengths is tested with increasing SNR, and the raw data is contaminated with zero-mean white Gaussian noise. The mean error $\bar{\chi }$ and the SD δ for distance and height are defined by:

(36) $${\bar{\chi }}_{\Theta }=\frac{1}{S}\overset{S}{\underset{s=1}{\sum }}\left[\chi_{\Theta }\left(s\right)\right],\;\delta_{\Theta }=\sqrt{\frac{1}{S}\overset{S}{\underset{s=1}{\sum }}{\left[\chi_{\Theta }\left(s\right)-{\bar{\chi }}_{\Theta }\right]}^{2}}\Theta =D,H$$

where S is the number of Monte Carlo, and Θ denotes the distance or height error. After soft assignment, each scatterer of the reconstructed target is assigned to the corresponding scatterer of the model:

(37) $$\chi_{D}\left(s\right)=\overset{K}{\underset{k=1}{\sum }}\varepsilon_{D}\left(s,k\right),\;\chi_{H}\left(s\right)=\overset{K}{\underset{k=1}{\sum }}\left|h_{m}\left(s,k\right)-h_{r}\left(s,k\right)\right|$$

where $\varepsilon_{D}\left(s,k\right)$ is the Euclidean distance between the scatterer of the model and corresponding scatterer of the reconstructed target. $h_{m}\left(s,k\right)$ and $h_{r}\left(s,k\right)$ are the height with respect to the image plane referring to the model and to the reconstructed target, respectively. The effective rotation vector can be obtained via:

(38) $${\bar{\chi }}_{ℵ}=\frac{1}{S}\overset{S}{\underset{s=1}{\sum }}\left|\hat{ℵ}\left(s\right)-ℵ\right|,\;\delta_{ℵ}=\sqrt{\frac{1}{S}\overset{S}{\underset{s=1}{\sum }}{\left[\hat{ℵ}\left(s\right)-E\left(\hat{ℵ}\left(s\right)\right)\right]}^{2}},ℵ=\Omega_{eff},\phi$$

where E( ) indicates the expectation. $\Omega_{eff}$ and ϕ are a priori known.

It is known that a shorter baseline will produce larger phase errors in measurement and decrease the accuracy of the height estimation [19]. Therefore, larger distance errors and height estimation errors will occur with a decreasing baseline. In Figure 7 and Figure 8, the curves of the mean distance error, mean height error, and their standard deviations follow a decreasing trend when the SNR or the baseline length increases, which is in accordance with theoretical predictions.

**Figure 9:**
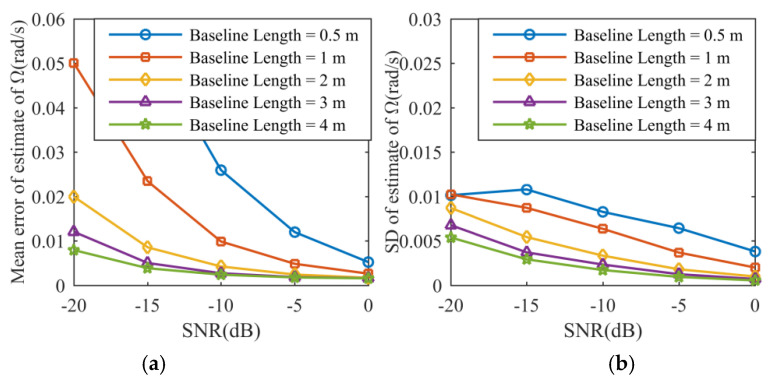
Simulation result. (**a**) Mean error of estimate of $\Omega_{eff}$. (**b**) Standard deviation of estimate of $\Omega_{eff}$.

**Figure 10:**
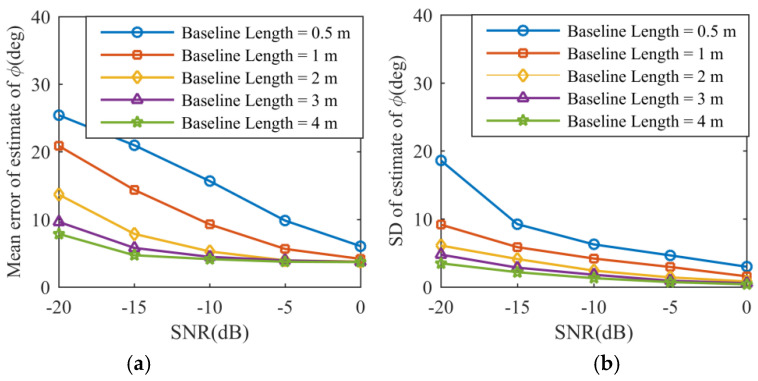
Simulation result. (**a**) Mean error of estimate of ϕ. (**b**) Standard deviation of estimate of ϕ.

Consistent with the previous conclusions, the mean errors and standard deviations of the estimates of $\Omega_{eff}$ and ϕ are given in Figure 9 and Figure 10, where the same decreasing pattern can be observed with increasing SNR and increasing baseline length. Compared to the method in [18], the same results can be obtained using the proposed method without any search processing or iteration procedures. Although the results above demonstrate that the longer baseline contributes to minimizing phase error measurement, there are conflicting requirements on the baseline length. According to Equation (19), the phase difference between the return echoes received by three antennas is a periodic function with period 2π. As a result, the altitude measurement is unambiguous if the phase difference exceeds 2π.

### 5.2. Verification with Complex Ship Model

**Figure 11:**
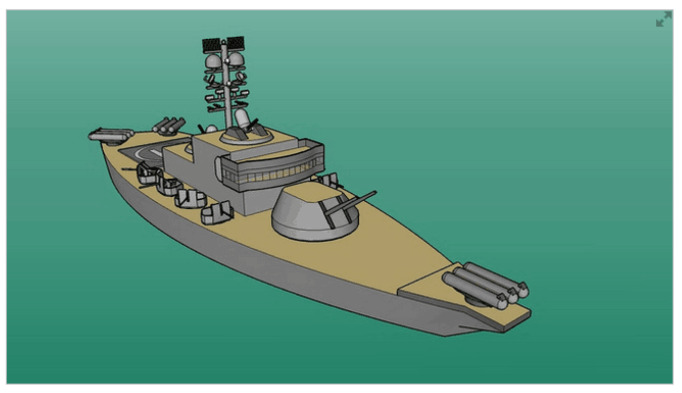
Ship target of interest.

In order to simulate a realistic echo return, the ship target model in Figure 11, whose dimensions are approximately 90(L)×27(W)×27(H)m, is shaped by 1274 scattering points depicted with their reflection coefficients and positions in the target coordinate system. The radar parameters are shown in Table 2.

**Figure 12:**
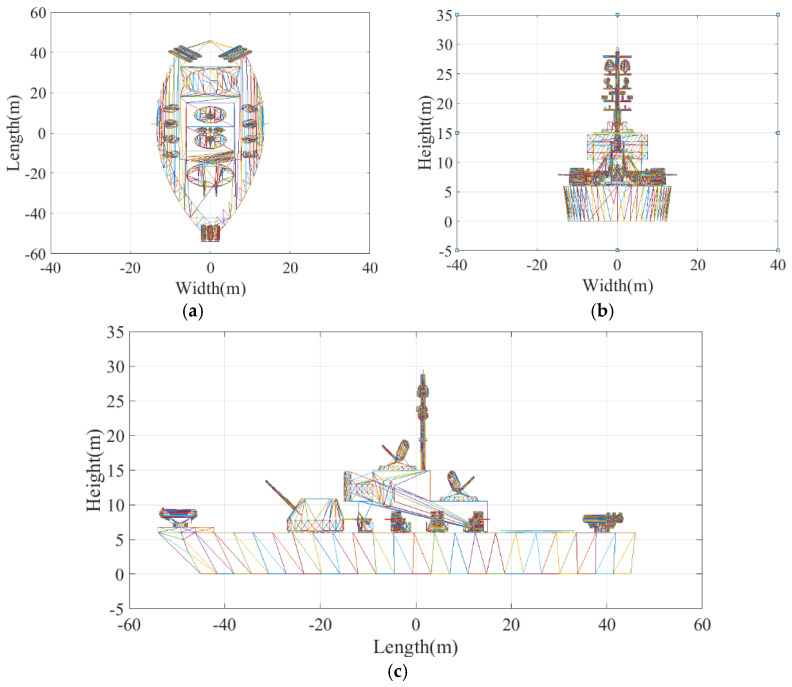
Three-dimensional tessellation ship target model. (**a**) Top view. (**b**) Front view. (**c**) Left view.

**Figure 13:**
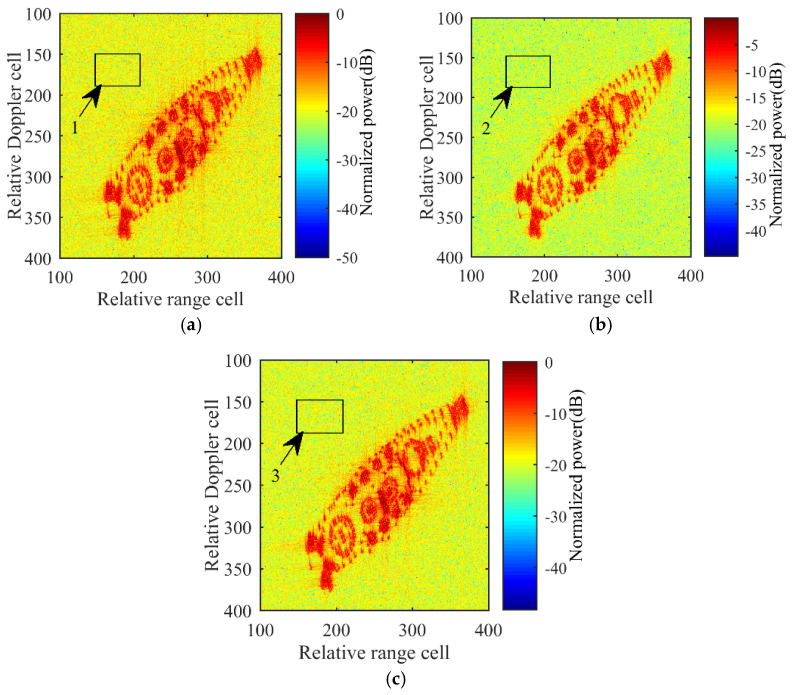
ISAR imaging results. (**a**) AV-Channel. (**b**) AC-Channel. (**c**) AH-Channel.

Similar to [14], this paper uses a standard tessellation language (STL) model shown in Figure 12 as an input to the simulation. Figure 13 shows the 2D ISAR images created via Equation (14) by processing the signal acquired from channels AV, AC, and AH, where the 2D ISAR images of the ship target are quite clearly visible. In Figure 13, the bounding box indicates a noise area of interest where the target is not present, which can be used to estimate the SNR of the bounding box to determine the power detection threshold. Based on the analysis and computation of the SNR of the bounding box in three ISAR images, values of $P_{\mathrm{noise},1}=-19.84\;\mathrm{dB}$, $P_{\mathrm{noise},2}=-19.79\;\mathrm{dB}$, and $P_{\mathrm{noise},3}=-19.82\;\mathrm{dB}$ are obtained.

**Figure 14:**
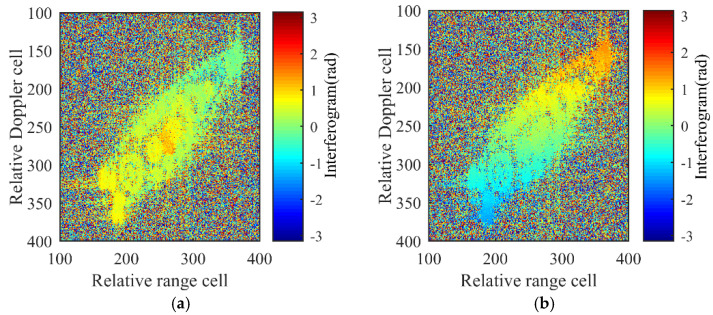
Interferograms. (**a**) AC–AV. (**b**) AC–AH.

The interferograms in Figure 14 are computed by processing the channel pairs AV–AC and AH-AC, where noise is present randomly, whereas the phase values of the target are scattered in an orderly manner and within the interval [−π,π], indicating that no phase wrapping occurs. The phenomena of the phase wrapping may occur when the phase differences in Equation (19) exceed 2π. From Equation (19), it is known that the interferometric phases are mainly determined by the target size, wavelength, and baseline length. Thus, users of the algorithm can set appropriate parameters to avoid this problem and refer to measures to deal with phase wrapping [18].

**Figure 15:**
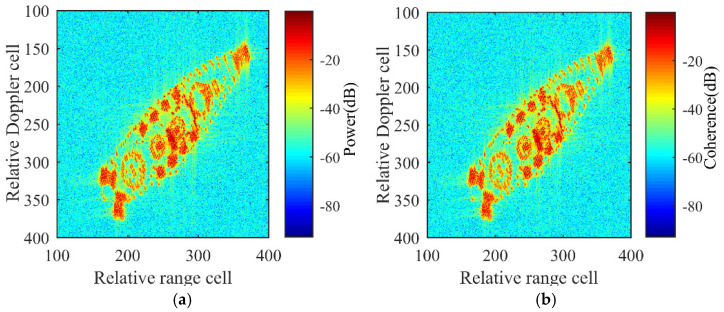
Coherence and power maps. (**a**) Power map. (**b**) Coherence map.

By observing Figure 13, the SNRs estimated from the three ISAR images are $P_{\mathrm{noise},1}=-19.84\;\mathrm{dB}$, $P_{\mathrm{noise},2}=-19.79\;\mathrm{dB}$, and $P_{\mathrm{noise},3}=-19.82\;\mathrm{dB}$, respectively. For three-channel coherence processing in this simulation, the threshold in image power depicted in Figure 15 can be obtained by calculating $P_{th}=P_{\mathrm{noise},1}+P_{\mathrm{noise},2}+P_{\mathrm{noise},3}+45\;\mathrm{dB}$. Thereby, the coherence threshold and power threshold can be set as $C_{th}\approx -15\;\mathrm{dB}$, $P_{th}\approx -15\;\mathrm{dB}$ to remove the low SNR resolution cells.

**Figure 16:**
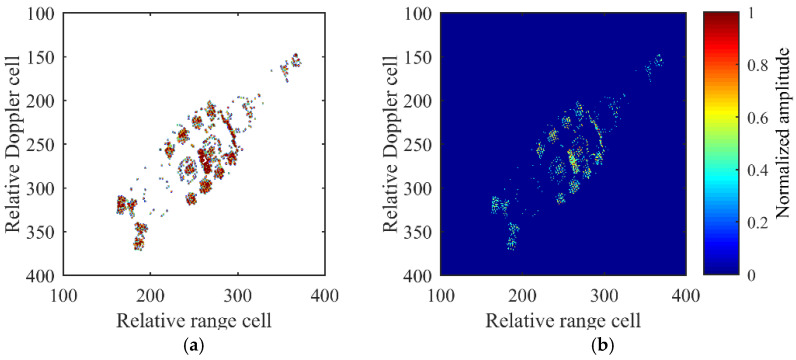
Detected mask and results after detection mask processing. (**a**) Detected mask. (**b**) 2D ISAR image received from the AC channel after detection processing.

The C3D detection mask from Equation (18) and 2D ISAR image received from the AC channel after detection mask processing are depicted in Figure 16a,b respectively. In Figure 16b, areas of no interest are removed and the effective information is preserved, after which the detected interferograms are passed to the 3D reconstruction process.

**Figure 17:**
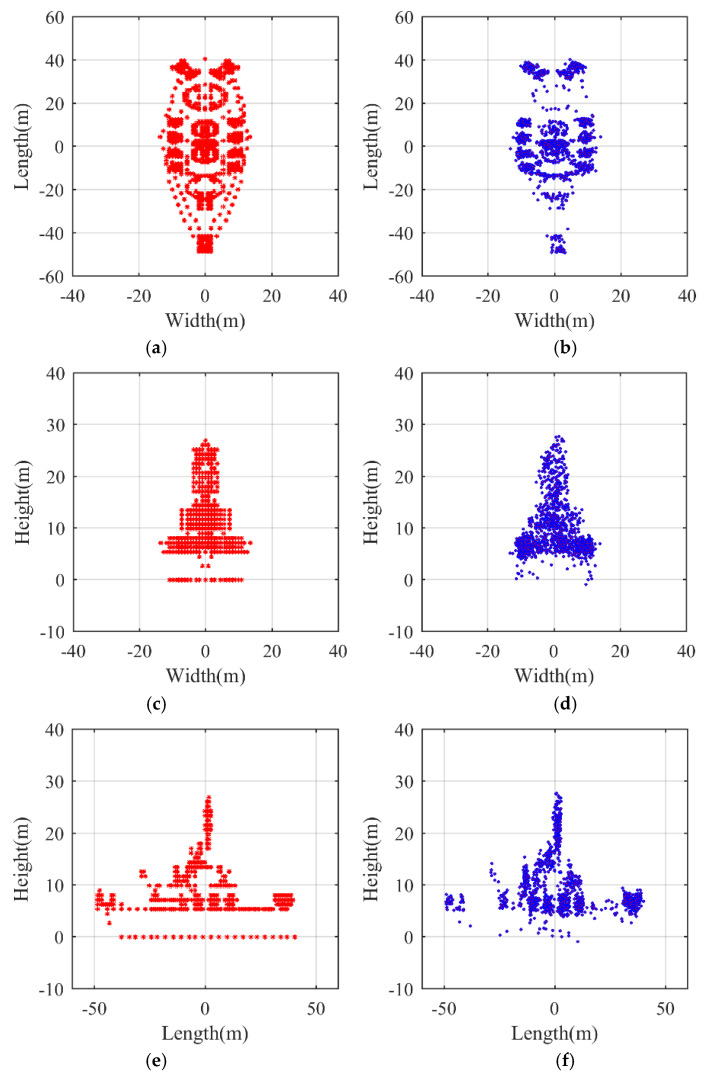
Model and reconstructed results using the proposed method. (**a**) Range–horizontal direction view of ship model. (**b**) Range–horizontal direction view of reconstructed result. (**c**) Range–vertical direction view of ship model. (**d**) Range–vertical direction view of reconstructed result. (**e**) Vertical–horizontal direction view of ship model. (**f**) Vertical–horizontal direction view of reconstructed result.

For comparison, the left column of Figure 17 shows the ideal model of the ship target in Figure 11, and the 3D InISAR reconstructed results are shown in the right column of Figure 17. The reconstructed ship target’s size almost matches the actual one depicted in Figure 17.

**Figure 18:**
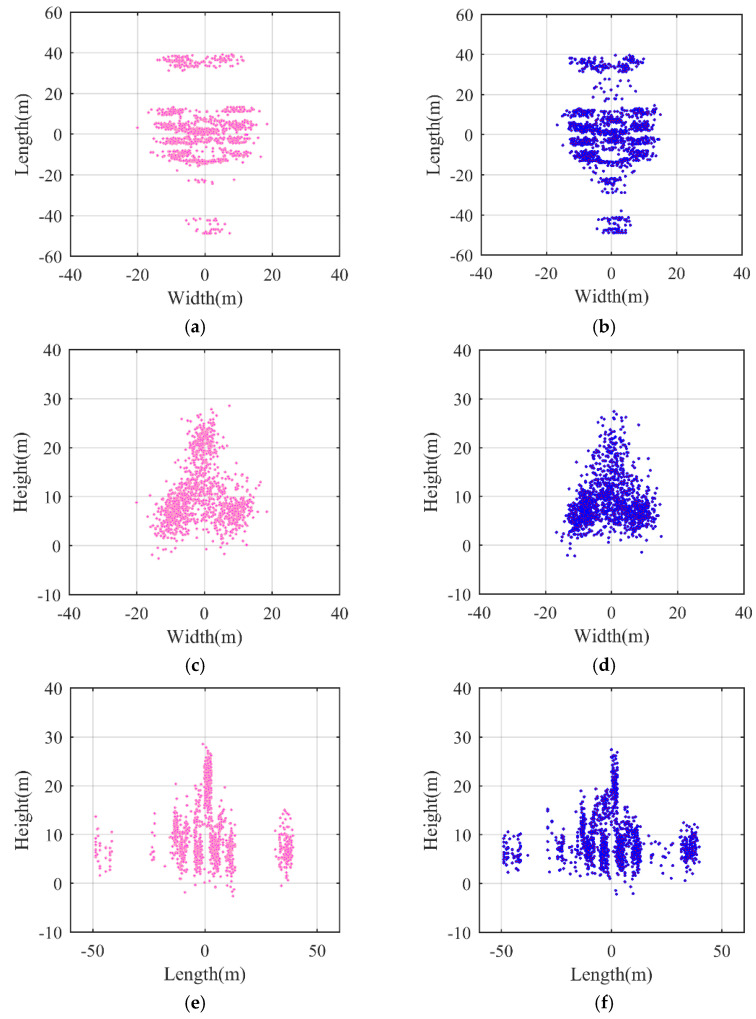
The reconstructed results using the method in [21] and the proposed method. (**a**) Range–horizontal direction view of result using the method in [21]. (**b**) Range–horizontal direction view of proposed method results. (**c**) Range–vertical direction view of result using the method in [21]. (**d**) Range–vertical direction view of proposed method results. (**e**) Vertical–horizontal direction view of result using the method in [21]. (**f**) Vertical–horizontal direction view of proposed method results.

Figure 17 verifies the viability of the proposed InISAR imaging algorithm. In Figure 18, an InISAR image based on the methods in [18,21] is chosen to compare with the imaging result based on the proposed method under the condition of *SNR* = 0 dB. The simulation times of the proposed 3D InISAR imaging method and the methods in [18] and [21] are 0.2 s and 12.6 s, demonstrating that our proposed algorithm is more efficient than the other methods. The reason is that the proposed C3D-based InISAR imaging algorithm employs holistic multiply–accumulate operations to extract the dominant scatterers instead of iterating or searching for pixels.

In order to quantitatively evaluate the performance of InISAR imaging, the relative mean error (RME) of the estimator is defined by:

(39) $$RME=\frac{1}{H}\sum_{h=1}^{H}\left|\frac{{\hat{ϒ}}_{h}-ϒ}{ϒ}\right|\mathrm{where}ϒ=\phi ,y_{1}ory_{3},$$

where $\hat{ϒ}$ and ϒ represent the estimated parameters and given parameters, respectively. H denotes the total number of extracted effective scattering points, and h denotes the hth effective scattering point. The tested input SNRs were SNR=[5,0,−5] dB, and 400 trials were performed for each SNR value.

After soft assignment, the RMEs of $\hat{\phi }$, ${\hat{y}}_{1}$-coordinate, and ${\hat{y}}_{3}$-coordinate using the proposed method and the method in [21] are shown in Table 3. As depicted, the RMEs of the estimated parameters with the proposed method in this paper are relatively small and within the acceptable range in a real scenario [46], which further demonstrates the effectiveness of our InISAR imaging algorithm. Theoretically, test losses may arise from approximation operations during the modeling of the echo signal and the extraction of the scattering center. Compared with the method in [21], the proposed method can acquire higher precision in parameter estimation and coordinate reconstruction. Due to the influence of error propagation in Equation (20), the RMEs of the reconstructed coordinates are larger than those of the rotational parameter estimation.

There are several artificial points in Figure 17 and Figure 18, which may be induced by noise or sidelobes of some scatterers. Therefore, in the future, more attention should be paid to reducing the effects of noise and sidelobes.

## 6. Conclusions

In this paper, a novel 3D target reconstruction algorithm based on an L-shape InISAR imaging system is presented, which makes use of Dechirp processing and interferometry. This imaging system consists of three antennas lying on two orthogonal baselines. The analytical expressions for estimating a target’s effective rotation vector and extracting 3D coordinates of the target are derived in detail. Moreover, a nonsearching detection algorithm, referred to as C3D, is introduced to remove the effects of noise and sidelobes while retaining areas of interest where the total power and coherence derived from the three channels are above thresholds.

Given the significance of pixel registration in 3D InISAR imaging, the paper also discusses and quantitatively analyzes the mismatching of two ISAR images and their influence on target reconstruction. In order to verify the robustness of the proposed algorithm, several experiments are conducted using simulated data in different scenarios. The simulation results are consistent with the theory that performance improves with increasing SNR and baseline length. At the same time, the present algorithm is proved to reconstruct the 3D coordinates of multi-scattering point targets with high accuracy and efficiency in the case of low signal-to-noise ratio, which is very suitable for practical applications.

Finally, the application and effectiveness of our InISAR imaging algorithm is validated by means of a simulated ship, with satisfactory results.

## Figures and Tables

**Table 1:** Simulation Parameters—Airplane.

Carrier Frequency	10 GHz	Baseline Length	$\begin{array}{c} \begin{array}{c} dv\;=\;0.5\;m\\ dh\;=\;0.5\;m \end{array}\}\mathrm{in}\;\mathrm{Figure}\;5\\ \begin{array}{c} dv\;=\;4.0\;m\\ dh\;=\;4.0\;m \end{array}\}\mathrm{in}\;\mathrm{Figure}\;6 \end{array}$
Fs	200 MHz	Radar-target distance	10 km
Bandwidth	200 MHz	Target velocity	60 m/s
Pulsewidth	2.56 us	SNR	0 dB
$T_{obs}$	0.5 s	Pitch/Roll/Yaw amplitude (${}^{\circ }$)	[1,2,10]
PRF	512 Hz	Pitch/Roll/Yaw period (s)	[5,8,9]

**Table 2:** Simulation Parameters—Ship.

Carrier Frequency	10 GHz	Baseline Length	$\begin{array}{c} dv\;=\;2m\\ dh\;=\;2m \end{array}$
Fs	200 MHz	Radar-target distance	16 km
Bandwidth	200 MHz	Target velocity	30 m/s
Pulsewidth	2.56 us	SNR	10 dB
$T_{obs}$	1 s	Pitch/Roll/Yaw amplitude (${}^{\circ }$)	[1,1,5]
PRF	512 Hz	Pitch/Roll/Yaw period (s)	[6,8,10]

**Table 3:** Reconstruction performance comparison.

RME (%)	5 dB		0 dB		−5 dB	
	Proposed	Method in [21]	Proposed	Method in [21]	Proposed	Method in [21]
$\hat{\phi }$	0.42	0.54	1.08	1.61	2.89	4.63
${\hat{y}}_{1}$	12.21	12.90	15.09	18.09	21.28	28.61
${\hat{y}}_{3}$	4.62	6.83	6.38	10.60	10.20	16.37

## Data Availability

Not applicable.
